# The B.M.A. Representative Meeting

**Published:** 1918-08-24

**Authors:** 


### The Hospital

The Workers' Newspaper of Administrative Medicine and Institutional Life, Administration, National Insurance and Health.

No. 1681, Vol. LXIV. SATURDAY, AUGUST 24, 1918. PRICE TWOPENCE

### MEDICAL OPINION AND ORGANISATION.

The recent annual meeting of the British Medical Association has afforded an opportunity for the expression of the opinion of the profession on various topics of immediate or future importance. Possibly the Association does not speak with such a full measure of authority as its officials imagine it to possess, yet not less true is it that the Representative Body unquestionably voices a considerable mass of professional judgment, and that its views are those widely held in professional circles. To judge from the composition of the Representative Meeting it must speak more particularly for the general practitioner, as the Representatives engaged in consulting practice are few and far between. In these days of universal democracy, however, this state of matters is hardly a source of weakness. At least it is not a source of weakness provided only that there is agreement on a common policy and determination on the measures necessary to give this policy a practical application. In the end it is with the general practitioners—that is, the enormous majority of the profession—that final decisions rest. Policies and proposals may be advanced by knights and baronets and colleges, but the adoption or refusal of them lies elsewhere. Whether the future will show a capacity to organise and to apply this potential force remains to be seen. History does not afford much encouragement in this direction, nor can it be said that the present shows any very hopeful development. But there is certainly a wide recognition of the probability that the profession is close to a critical point in its position and future, and on all hands there is affirmed the need for organisation. To cry out for organisation is one thing, to achieve it is another; and success of this latter order is still sadly to seek. There can be no doubt where the responsibility for failure—if failure occurs—will rest. It will rest with the profession itself. In the whole scheme of social life there is no body of workers which—union being granted—has so great a potential ability to fix the conditions of its service. The nation cannot do without the medical practitioner, nor can it replace him. Hence if the profession as a whole is united it can fix its own terms. But, of course, it needs an organ through which its will can be expressed and by which its decision can be applied, and so far this cannot be said to have been secured. The universities and the colleges are quite detached from the general body of the profession and have no authority to speak on its behalf. The B.M.A. no doubt has a large membership and a democratic organisation, but many practitioners stand outside its ranks, and not a few of its members are wholly passive and inert. Of the smaller organisations one frankly seeks to make the profession a trades union, but even its advocates can hardly hope to establish this principle in time to deal with immediate issues. If salvation is to be found it must be by the adaptation of existing machinery and by the decision of all the members of the profession to give loyal support to a practical and constructive policy.

One item of the business of the recent Representative Meeting affords striking proof of the ability of the profession to enforce its demands even on a powerful department of Government. It is well known that for some time there has been great dissatisfaction with the conditions imposed on the Indian Medical Service, and that remonstrances have not been well received by the constituted authorities. Recently there has been more determined action, and the powers that be have had to learn that the medical schools have resolved to advise students not to join the Indian Medical Service. The result is seen in a message from the Secretary of State to the Representative Meeting and conveying a promise that grievances shall be redressed and satisfactory conditions of pay and service and opportunity be established. The promise has yet to take practical shape, but of its value there can be no doubt, and equally there can be no doubt that the guarantee which it contains springs from a perception of the truth that the profession is in a position to fix the conditions on which its members will undertake duty in the Indian Medical Service. Unless these conditions are granted recruits for the Service will either entirely disappear or will be greatly restricted alike in quality and in quantity. The lesson is a plain one. Let the profession put themselves in a similar position in relation to all other public Services—and if united they can easily do this—and a parallel result will follow. No doubt it would be more gracious and more welcome if justice and fair consideration could be secured without threats and reprisals. But we have to take the world as we find it, and experience teaches plainly that good and generous service is most likely to receive recognition when it is associated with the ability to withdraw its values. Many men and most public bodies are unmoved by loyal and efficient co-operation unless they are compelled to feel that this co-operation may not endure. Such a conclusion may have a cynical quality, but will anyone deny that it affords a wise rule of conduct?

There is, we fancy, no danger of the profession forgetting its altruistic tendencies and traditions, and we are far from suggesting that it should do so. Hence we have no words other than those of congratulation to offer to the Representative Meeting on its decision to enter no protest against the conscription of medical men up to fifty-six years of age, while for the rest of the community the claim ceases five years earlier. We view in the same spirit the expressed wish that the B.M.A. should continue to pay all the expenses of the Central Medical War Committee, though essentially the work of this committee is Government work and though it involves much personal sacrifice to most of its members. Whether on the battlefield or at home the profession has no need to be ashamed of its war record. From one of the recent discussions it appears that some doctors believe that if only they could get a few of their number into the House of Commons all would be well. It is an ingenuous creed. In our view they have an infinitely stronger apparatus if only they will use it. Let them think out clearly what they want, and then present a united front.

